# Glucocorticoid therapy for acute interstitial nephritis in a patient with diabetic nephropathy: A case report

**DOI:** 10.1097/MD.0000000000051477

**Published:** 2026-11-06

**Authors:** Yaguang Xu, Tianshu Wang, Hongjiao Zhang, Jie Lv

**Affiliations:** a Department of Nephrology, The Second People’s Hospital of Liaocheng, Linqing, Shandong Province, China.

**Keywords:** acute interstitial nephritis, case report, diabetic nephropathy, glucocorticoids, kidney biopsy

## Abstract

**Rationale:**

Acute interstitial nephritis (AIN) can cause acute kidney injury. Diabetic nephropathy (DN) is a chronic microvascular complication of diabetes. The coexistence of these diseases complicates diagnosis and treatment.

**Patient concerns:**

A 41-year-old man with diabetes and hypertension presented with dizziness on 4 January 2025. His renal function was normal 7 months previously, but his sCr was 258 μmol/L immediately before admission and peaked at 299 μmol/L after admission. The patient reported use of an unidentified herbal decoction during the previous month.

**Diagnoses:**

A biopsy showed acute tubular injury (70% of tubules), mild tubulitis (<5% of tubules), and interstitial inflammatory infiltration (~10% of cortex), consistent with AIN. The glomerular findings met the 2010 Renal Pathology Society criteria for class IIa DN. There was no infection or systemic autoimmune disease. The herbal decoction was a possible trigger.

**Interventions:**

Prednisone was started on 15 January 2025 (0.52 mg/kg/d), followed by tapering and discontinuation. Repaglinide and insulin were used for glycemic control. Dapagliflozin was added at follow-up for glycemic normalization and renoprotection. Hypertension was treated, but no adjustments were required after glucocorticoid initiation.

**Outcomes:**

At discharge, the sCr was 194 μmol/L. At the follow-up (March 19, 2026), there were improvements in the sCr, eGFR, blood urea nitrogen, HbA1c, and 24-h urinary protein, and these clinical parameters were about the same at the last follow-up (April 9, 2026). The antidiabetic regimen was continued unchanged, and the most recent HbA1c was 5.6% (March 19, 2026). The patient developed corticosteroid-associated hyperglycemia and insomnia. The hyperglycemia was successfully managed with insulin, and the insomnia improved during corticosteroid tapering. Dapagliflozin was subsequently added for glycemic control and kidney protection. No infection was recorded.

**Lessons:**

Kidney biopsy should be considered when a patient with diabetes develops acute kidney dysfunction because AIN can coexist with DN. This patient’s kidney function improved after initiation of prednisone, but antihypertensive therapy was intensified before prednisone treatment. Glycemic therapy was then modified, and the possible offending herbal decoction was previously discontinued. Although we only examined a single patient with AIN and DN, glucocorticoid therapy may have contributed to the resolution of AIN.

## 1. Introduction

Acute interstitial nephritis (AIN) is characterized by inflammatory-cell infiltration of the renal interstitium with tubular injury and dysfunction.^[1]^ Medications are the most common causes, followed by infections and autoimmune disorders.^[2]^ Diabetic nephropathy (DN), a chronic microvascular complication of diabetes, is characterized pathologically by glomerular basement membrane (GBM) thickening and mesangial expansion.^[3,4]^

When AIN and DN coexist, the clinical presentation may be nonspecific, and kidney biopsy may be required to distinguish an acute potentially reversible lesion from chronic diabetic injury.^[2]^ Glucocorticoids may be given to selected patients with AIN, but this may worsen hyperglycemia and complicate the management of diabetes.^[5]^ We report a patient with biopsy-proven AIN superimposed on Renal Pathology Society (RPS) class IIa DN whose kidney function improved following tapered glucocorticoid therapy, intensified antihypertensive treatment, and subsequent antihyperglycemic adjustments. This report was prepared in accordance with the CARE reporting guideline.

## 2. Case presentation

### 2.1. Presentation and history

A 41-year-old man was admitted on 4 January 2025 after one day of dizziness. A laboratory test on 3 January showed the serum creatinine (sCr) was 258 μmol/L, the estimated glomerular filtration rate (eGFR) was 25.49 mL/min/1.73 m^2^, and blood urea nitrogen (BUN) was 13.49 mmol/L. At a health examination on June 17, 2024, the sCr was 82 μmol/L, the urinalysis results were normal (negative for urinary protein and occult blood), and the eGFR was 102.64 mL/min/1.73 m^2^. His medical history included type 2 diabetes mellitus (T2DM) for 2 years and hypertension for 6 months. Neither parent had diabetes. His father had a long-standing history of asthma, and his mother had long-standing hypertension and a history of cerebral infarction.

He was taking metformin (0.5 g once daily) for approximately 2 years without regular glucose monitoring. Metformin was discontinued upon admission, and repaglinide (1 mg 3 times daily) was started. He was also prescribed amlodipine (5 mg daily) for approximately 6 months but reported taking it only about 4 days per week. Amlodipine (5 mg daily) was continued in the hospital; metoprolol succinate (23.75 mg daily) was added on 4 January; and terazosin (2 mg nightly) was added on 6 January and increased to 2 mg twice daily on 8 January.

During detailed questioning, he reported taking an unidentified herbal decoction from a local traditional-medicine clinic twice daily for 15 days during the month before presentation because of dissatisfaction with his sexual function. He denied use of other agents (antibiotics, nonsteroidal anti-inflammatory drugs [NSAIDs], proton pump inhibitors [PPIs], dietary supplements, iodinated contrast media, or other herbal or traditional medicines) during the preceding 3 months. He also reported no recent fever, rash, arthralgia, upper respiratory infection, or urinary tract infection.

At admission, his blood pressure was 163/117 mm Hg and there was minor pitting edema in both lower extremities.

### 2.2. Laboratory and imaging findings

On 5 January, the sCr was 299 μmol/L, eGFR was 21.33 mL/min/1.73 m^2^, BUN was 14.88 mmol/L, urinary albumin-to-creatinine ratio was 56.58 mg/g, and glycated hemoglobin (HbA1c) was 7.7%. In addition, the white-cell count was 10.22 × 10^9^/L, with neutrophils of 6.11 × 10^9^/L (57.9%), lymphocytes of 2.47 × 10^9^/L (24.2%), and eosinophils of 0.53 × 10^9^/L (5.2%). The urinalysis was negative for protein and occult blood; there were 2 white blood cells per high-power field, but no white-cell casts, red-cell casts, or eosinophils on routine microscopy. A dedicated urine eosinophil test was not performed.

Urine output was 1500 to 2000 mL/day soon after admission, with nocturia (three times nightly), but no oliguria. After prednisone was started, the urine output increased temporally to a maximum of 3500 mL/day, and was approximately 2500 mL/day at discharge.

The patient remained afebrile (36.2–36.8°C). The C-reactive protein was 2.67 mg/L, the procalcitonin was below 0.1 ng/mL, and the erythrocyte sedimentation rate was 8 mm/h. The tests for hepatitis B, hepatitis C, human immunodeficiency virus, and syphilis were negative, as were the tests for antinuclear antibodies, anti-double-stranded DNA antibodies, and antineutrophil cytoplasmic antibodies (ANCA). The level of C3 was 1.46 g/L, and the level of C4 was 0.35 g/L. The levels of serum IgG, IgA, IgM, and IgE were 10.1 g/L, 1.05 g/L, 0.614 g/L, and 227 IU/mL, respectively. Serum immunofixation electrophoresis showed no abnormal bands. Serum free light chains were not measured.

Renal ultrasonography showed mildly enlarged kidneys (left: 13.1 × 6.2 cm, right: 12.5 × 6.0 cm), with mildly increased cortical echogenicity and indistinct corticomedullary differentiation. There was no evidence of hydronephrosis, calculus, or cyst, and renal Doppler ultrasonography showed normal bilateral renal blood flow.

### 2.3. Kidney biopsy

A kidney biopsy was performed on 10 January 2025. Light microscopy of 10 glomeruli showed global sclerosis in 1 glomerulus and mild diffuse expansion of mesangial cells and matrix in the other glomeruli, with mild GBM thickening and no nodular (Kimmelstiel-Wilson) sclerosis (Fig. 1). There were no spike-like structures, subepithelial or subendothelial deposits, parietal-cell hyperplasia, or crescents.

**Figure 1.**
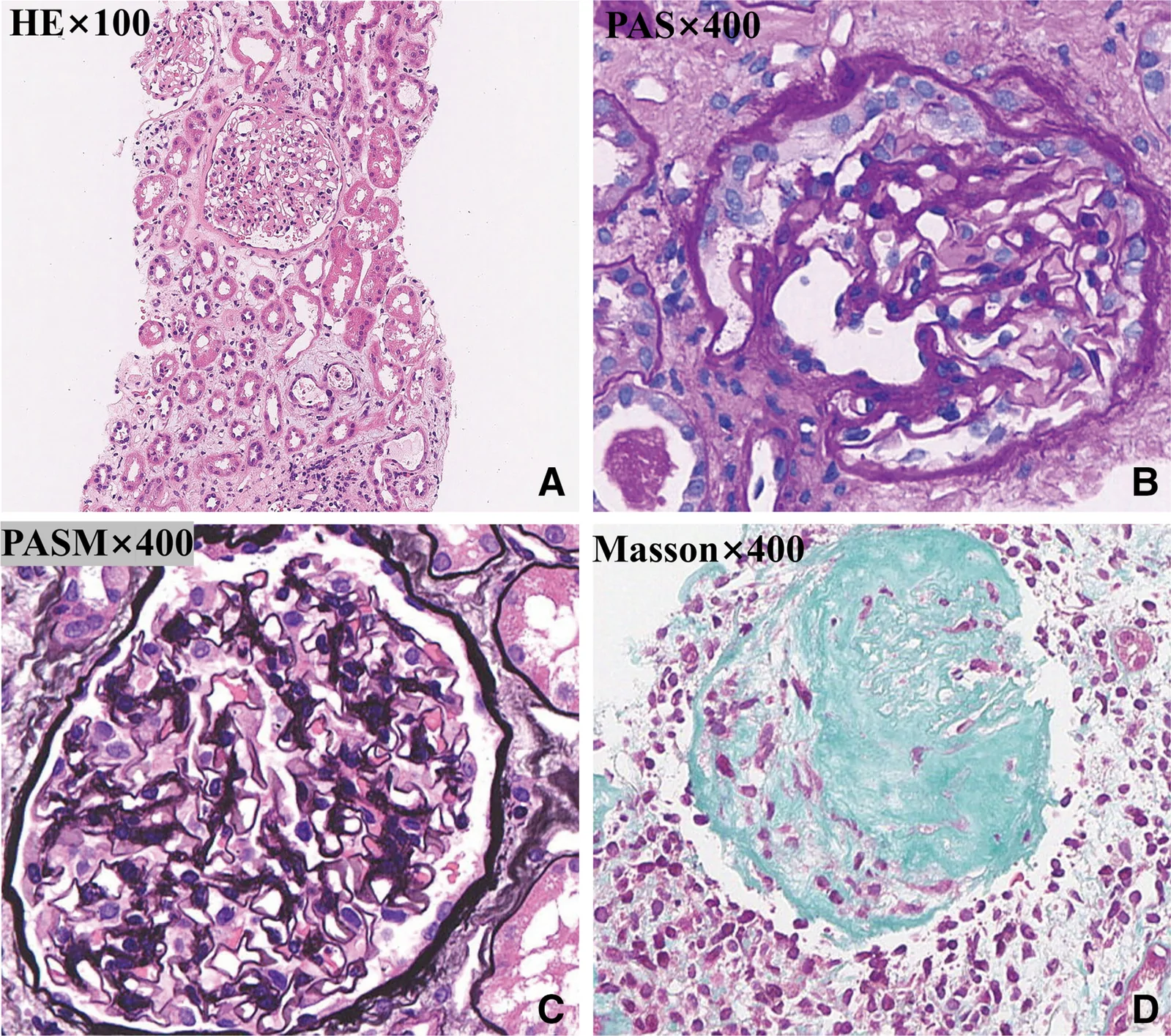
Light microscopy of the kidney biopsy. (A) Hematoxylin-eosin staining (×100), showing interstitial edema with focal inflammatory-cell infiltration (predominantly lymphocytes with scattered plasma cells), mild fibrosis, tubular dilatation, loss of brush borders, focal tubular atrophy, and combined IFTA of approximately 10%. (B) Periodic acid-Schiff staining (×400), showing mild diffuse proliferation of glomerular mesangial cells and matrix. (C) Periodic acid-silver methenamine staining (×400), showing mild GBM thickening without spike-like structures, subepithelial or subendothelial fuchsinophilic deposits, parietal-cell proliferation, or crescents. (D) Masson trichrome staining (×400), showing one globally sclerotic glomerulus with surrounding inflammatory-cell infiltration.

Examination of the renal tubular epithelial cells showed granular and vacuolar degeneration, loss of brush borders, epithelial simplification, and widened lumina, with acute tubular injury in approximately 70% of the tubules. There was mild tubulitis in <5% of the tubules. The interstitium was edematous, and there was inflammatory-cell infiltration in approximately 10% of the cortical area, which was composed predominantly of lymphocytes with scattered plasma cells and approximately 5% eosinophils. There was combined interstitial fibrosis and tubular atrophy in approximately 10% of the cortex (RPS interstitial fibrosis and tubular atrophy [IFTA] score 1). There was no arteriolar hyalinosis (score 0), and arterial intimal thickening did not exceed medial thickness (arteriosclerosis score 1).

Immunofluorescence staining was negative for IgG, IgA, C3, C1q, fibrinogen, κ light chain, and λ light chain. The glomerular mesangial region showed segmental weak (±) IgM positivity, and albumin (ALB) reabsorption droplets were present in the renal tubular epithelial cells (Fig. 2).

**Figure 2.**
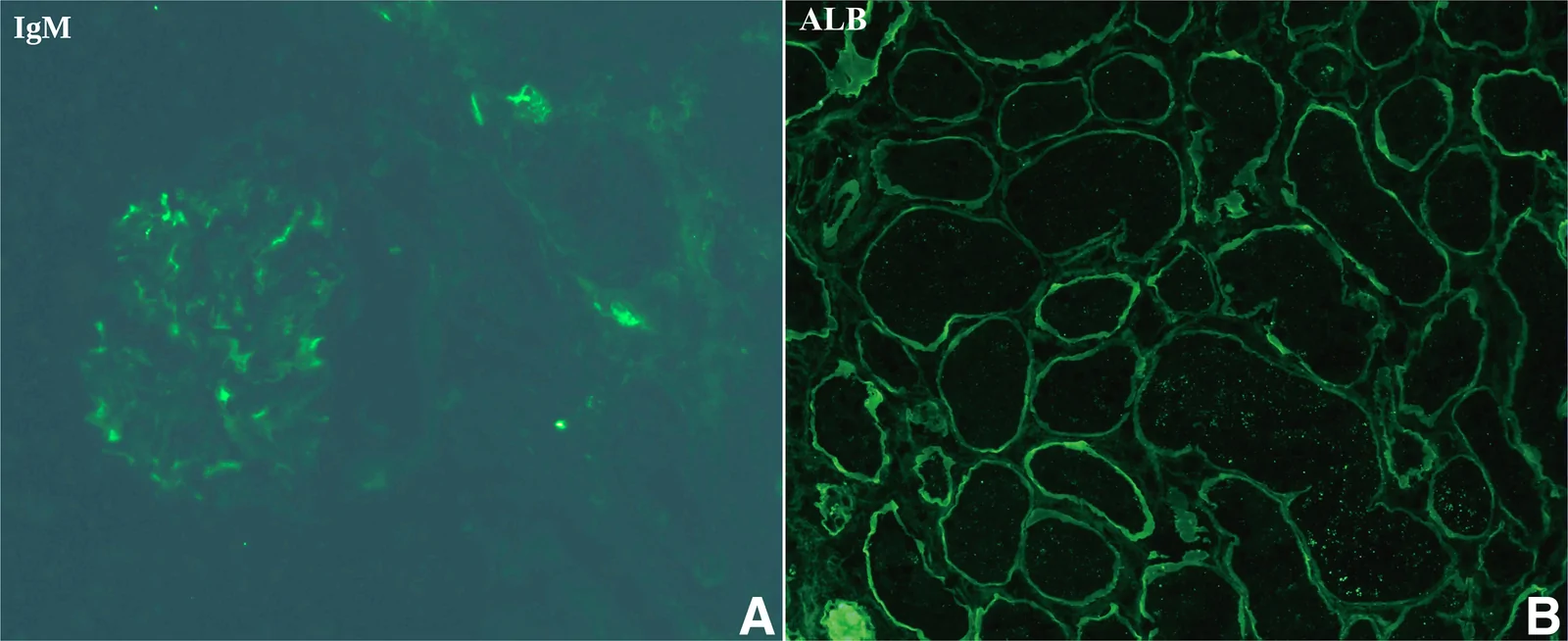
Immunofluorescence microscopy of the kidney biopsy (×200). (A) IgM staining, showing segmental weak (±) positivity in the glomerular mesangial region. (B) Albumin staining, showing reabsorption droplets in the cytoplasm of renal tubular epithelial cells.

Electron microscopy showed segmental wrinkling, subendothelial-space expansion, and mild homogeneous GBM thickening (range 300–620 nm; mean 500 nm; Fig. 3). There was segmental podocyte foot-process effacement in <25% of the capillary surface. Mesangial cells and matrix were mildly increased, but there were no electron-dense deposits. Some tubular epithelial cells were degenerated and detached, and there was mild interstitial edema and scattered inflammatory cells.

**Figure 3.**
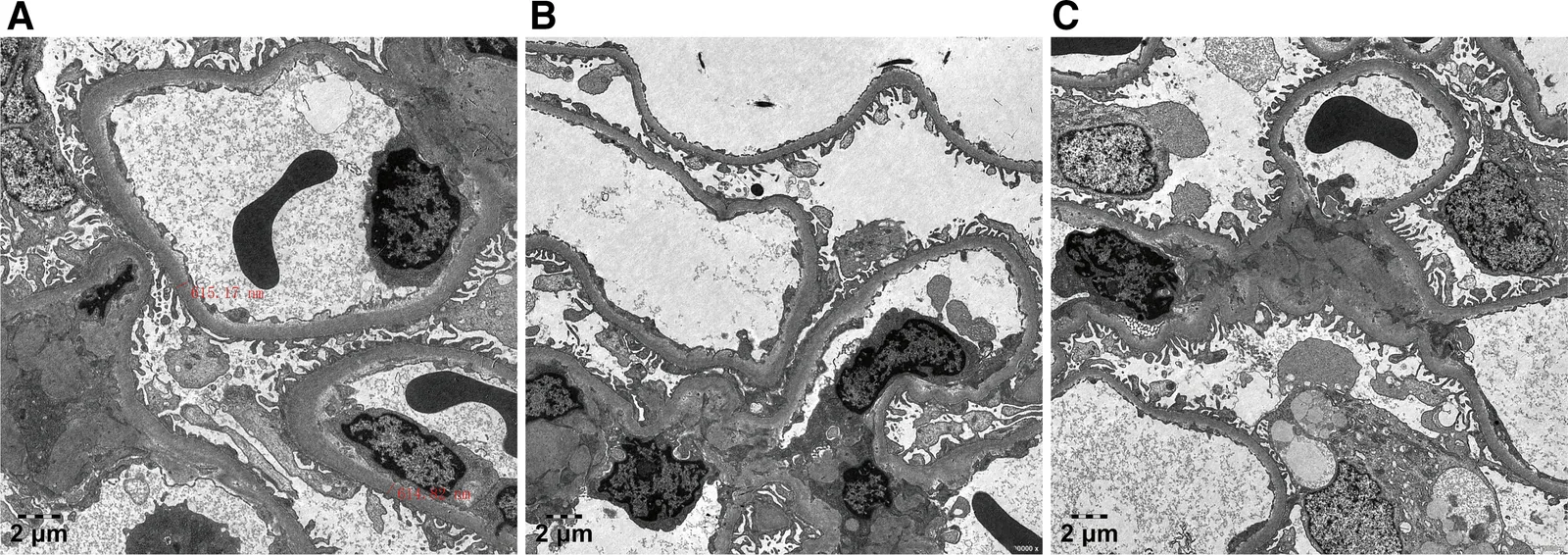
Transmission electron microscopy of the kidney biopsy. (A) × 6000, showing mild homogeneous GBM thickening (615 nm in the illustrated field). (B) × 5000, showing mild homogeneous GBM thickening without electron-dense deposits. (C) × 5000, showing mild homogeneous GBM thickening with segmental podocyte foot-process effacement of <25% of the capillary surface.

According to the 2010 RPS classification of DN,^[6]^ global sclerosis in 1 of 10 glomeruli was below the 50% threshold for class IV, nodular sclerosis was absent (excluding class III), and mesangial expansion exceeded isolated GBM thickening (excluding class I). The glomerular lesion was therefore classified as class IIa DN (mild mesangial expansion). The tubulointerstitial and vascular lesions were scored separately, as described above.

### 2.4. Diagnosis

The acute tubulointerstitial inflammatory infiltrate and tubular injury were diagnostic of AIN. The recent 15-day exposure to an unidentified herbal decoction was the only known plausible trigger. Because the composition of this decoction was unavailable and causality could not be established, we considered the AIN as possibly herb-associated rather than definitively drug-induced. The infection and systemic autoimmune evaluations were unrevealing. The concurrent glomerular changes were consistent with RPS class IIa DN.

### 2.5. Treatment and follow-up

The patient was 165 cm tall and weighed 58 kg (BMI, 21.3). Prednisone acetate was started on January 15, 2025 at 30 mg/day (0.52 mg/kg/day) and maintained for 30 days. The dose was reduced to 20 mg/day on 14 February, 15 mg/day on 18 March, 10 mg/day on 15 April, and 5 mg/day on 13 May, and discontinued on 17 June 2025. The medical record documented these dose changes at follow-up, but a formal adherence instrument was not used. A moderate initial dose was selected because of coexisting diabetes and the risk of corticosteroid-associated hyperglycemia.^[7]^

We initially managed the hyperglycemia with repaglinide (1 mg three times daily). At discharge on 20 January, the fasting blood glucose had risen to 8.79 mmol/L and was considered corticosteroid-associated. We therefore prescribed premixed human insulin 30/70 (Novolin 30R) at 12 U before breakfast and 10 U before dinner, increased the dose to 14 U/12 U on 14 February, decreased the dose to 12 U/10 U on 18 March, and decreased the dose again to 8 U/6 U at the 19 March 2026 follow-up. At this follow-up, we added dapagliflozin (10 mg once daily) for glycemic control and renoprotection because of the DN. At the last follow-up (April 9, 2026), the sCr had mildly increased to 121 μmol/L (an increase <30%), and Novolin 30R (8 U/6 U) and dapagliflozin (10 mg daily) were continued. Insomnia also occurred during corticosteroid treatment but decreased during corticosteroid tapering. There was no infection, new edema, corticosteroid-related blood-pressure deterioration, or skeletal adverse event. In addition, no adjustments to antihypertensive therapy were required after glucocorticoid initiation, but the antihyperglycemic treatment was modified.

At discharge, the sCr had decreased to 194 μmol/L. At the follow-up on 19 March 2026, the sCr was 107 μmol/L, eGFR was 73.37 mL/min/1.73 m^2^, BUN was 8.56 mmol/L, fasting blood glucose (FBG) was 5.68 mmol/L, HbA1c was 5.6%, and 24-h urinary protein was 162 mg. At the last follow-up (9 April 2026), the sCr was 121 μmol/L, eGFR was 63.23 mL/min/1.73 m^2^, BUN was 8.69 mmol/L, FBG was 5.02 mmol/L, and 24-h urinary protein was 122 mg. We continued the antidiabetic regimen, and the most recent HbA1c was 5.6% (19 March 2026). The clinical timeline is shown in Table 1.

**Table 1:** Timeline of clinical and laboratory parameters from the most recent pre-illness measurement to the last follow-up.

Event (date)	sCr (μmol/L)	eGFR (mL/min/1.73 m^2^)	BUN (mmol/L)	Urinary protein/UACR	FBG (mmol/L)	HbA1c (%)	BP (mm Hg)	Prednisone (mg/day)
Pre-illness examination (17 Jun 2024)	82	102.64	6.75	Urinalysis normal; protein and occult blood negative	NR	NR	NR	-
Pre-admission test (3 Jan 2025)	258	25.49	13.49	NR	7.45	NR	155/102	-
Admission assessment^*^ (4–5 Jan 2025)	299	21.33	14.88	UACR 56.58 mg/g	NR	7.7	163/117	-
7 Jan 2025	NR	NR	NR	24-h protein 233 mg	6.51	NR	152/98	-
9 Jan 2025	290	22.13	16.00	NR	NR	NR	141/93	-
Kidney biopsy (10 Jan 2025)	272	23.92	15.78	NR	6.02	NR	137/92	-
Prednisone started (15 Jan 2025)	NR	NR	NR	NR	NR	NR	135/89	30
Discharge (20 Jan 2025)	194	35.99	12.53	24-h protein 261 mg	8.79	NR	139/92	30
Follow-up (14 Feb 2025)	162	44.75	11.27	24-h protein 205 mg	8.25	7.9	136/95	20
Follow-up (18 Mar 2025)	156	46.84	10.92	24-h protein 226 mg	6.15	NR	141/88	15
Follow-up (15 Apr 2025)	133	56.80	9.20	NR	5.91	NR	128/81	10
Follow-up (13 May 2025)	128	59.49	8.85	24-h protein 195 mg	5.67	6.1	131/86	5
Prednisone discontinued (17 Jun 2025)	125	61.22	8.63	24-h protein 157 mg	5.74	NR	125/83	0
Follow-up (21 Aug 2025)	120	64.32	9.02	NR	5.43	NR	121/87	0
Follow-up (30 Oct 2025)	115	67.72	8.75	24-h protein 139 mg	5.52	5.8	129/91	0
Follow-up (19 Mar 2026)	107	73.37	8.56	24-h protein 162 mg	5.68	5.6	132/87	0
Last follow-up (9 Apr 2026)	121	63.23	8.69	24-h protein 122 mg	5.02	NR	128/85	0

NR = not recorded in the clinical data supplied.

* Admission blood pressure was measured on 4 January 2025; admission sCr, eGFR, BUN, and UACR were measured on 5 January 2025.

## 3. Discussion

AIN and DN have distinct pathophysiologic mechanisms, but their coexistence can make clinical diagnosis and treatment difficult. The biopsy in this patient identified both an acute tubulointerstitial process and chronic diabetic glomerular injury, allowing the acute and chronic components to be considered separately.

Inflammatory-cell infiltration of the interstitium and tubular injury are central features of AIN.^[8]^ Fever, rash, and eosinophilia are inconsistent and nonspecific findings.^[2]^ Management includes identifying and withdrawing a potential trigger and providing supportive care. Glucocorticoids are commonly used in selected cases of drug-induced or otherwise immune-mediated AIN, particularly when kidney function does not improve after trigger withdrawal.^[5]^ Their anti-inflammatory effects may reduce interstitial edema and limit progression to fibrosis.^[9]^

DN is a chronic microvascular disease characterized by GBM thickening, mesangial expansion, and progressive glomerulosclerosis.^[10]^ Long-term control of blood glucose and blood pressure is essential to slow progression.^[11]^ In patients with diabetes, an abrupt rise in sCr should prompt consideration of a superimposed acute lesion, including AIN.^[12–14]^

This patient used an unidentified herbal decoction for 15 days in the month before presentation because of dissatisfaction with his sexual function. Several cases of AIN or acute tubulointerstitial nephritis associated with herbal or folk medicines have been reported, including cases linked to Achyranthes japonica, Corni Fructus, and other herbal preparations.^[15–18]^ In the present patient, the temporal relationship between herbal-decoction exposure and kidney dysfunction, together with the absence of another recent plausible exposure and the unrevealing infection and autoimmune evaluations, makes the herbal decoction a plausible trigger. However, because the preparation’s composition was unavailable and causality could not otherwise be confirmed, a causal relationship cannot be established.

We initiated prednisone because the biopsy showed active tubulointerstitial inflammation and extensive acute tubular injury. Notably, the sCr had already decreased from 299 to 272 μmol/L before prednisone was initiated on January 15, 2025, indicating that kidney function had begun to improve before glucocorticoid therapy and further limiting causal attribution to prednisone. After prednisone initiation, the sCr decreased further to 194 μmol/L at discharge and to 107 μmol/L (March 19, 2026 follow-up) and 121 μmol/L (last follow-up on April 9, 2026). This mild increase of sCr was <30%. The subsequent course is compatible with a possible contribution from prednisone-mediated resolution of the acute inflammatory process, but the pretreatment improvement and other concurrent changes prevent causal attribution to prednisone. We initiated dapagliflozin (10 mg daily) at the March 19, 2026 visit for glycemic normalization and renoprotection in the context of established DN. The mild sCr increase at the subsequent visit is consistent with the known hemodynamic effect of sodium-glucose transport protein 2 inhibitors on intraglomerular pressure, and we continued this regimen.

## 4. Alternative and contributory explanations for improvement

The patient experienced several concurrent changes. The severe hypertension was corrected, which may have reduced pressure-related hemodynamic injury. The previously inconsistent antihypertensive therapy and insufficiently monitored diabetes were addressed with regular multidrug treatment. The possible offending herbal preparation had already been stopped, so spontaneous improvement after withdrawal cannot be excluded. The effect of each of these factors cannot be determined by examination of a single uncontrolled case.

This case supports 4 practical considerations. First, kidney biopsy should be considered when a patient with diabetes has an acute and otherwise unexplained decline in kidney function. Second, an acute tubulointerstitial lesion and chronic diabetic lesion should be managed separately. Third, glucocorticoid-associated hyperglycemia, infection, blood-pressure elevation, and other adverse effects require close monitoring and drug tapering. Fourth, long-term follow-up is needed because sCr may remain above the pre-illness baseline even after substantial recovery.

## 5. Limitations

This report had several limitations. It describes one patient and therefore cannot establish the efficacy of glucocorticoid therapy for AIN in patients with DN. There was only one pre-illness (June 17, 2024) measurement of sCr (82 μmol/L) and eGFR (102.64 mL/min/1.73 m^2^). There was no repeat biopsy. Prednisone was started after antihypertensive treatment had been intensified and after sCr had already declined from 299 to 272 μmol/L, further limiting causal attribution to glucocorticoid therapy; antihyperglycemic treatment was modified during follow-up after the possible trigger was withdrawn. The herbal preparation could not be identified, so attribution of disease to that herbal decoction agent remains uncertain. A formal assessment of adherence and a formal patient-perspective statement were not obtained.

## Author contributions

**Conceptualization:** Yaguang Xu.

**Data curation:** Yaguang Xu, Tianshu Wang.

**Formal analysis:** Yaguang Xu, Tianshu Wang.

**Validation:** Yaguang Xu, Jie Lv.

**Writing – original draft:** Yaguang Xu, Tianshu Wang.

**Resources:** Hongjiao Zhang, Jie Lv.

**Visualization:** Hongjiao Zhang, Jie Lv.

**Writing – review & editing:** Jie Lv.
